# Metabolism in the tumor microenvironment: implications for pathogenesis and therapeutics

**DOI:** 10.3389/fimmu.2025.1610255

**Published:** 2025-10-29

**Authors:** Jonas Aakre Wik, Emma Riiser Berge, Kristine Stromsnes, Bjørn Steen Skålhegg

**Affiliations:** ^1^ Hybrid Technology Hub - Centre of Excellence, Institute of Basic Medical Sciences, University of Oslo, Oslo, Norway; ^2^ Department of Immunology and Transfusion Medicine, Oslo University Hospital, Oslo, Norway; ^3^ Division for Molecular Nutrition, Institute of Basic Medical Sciences, University of Oslo, Oslo, Norway

**Keywords:** metabolism, cancer, T cells, macrophages, immunotherapy, tumor microenvironment, angiogenesis

## Abstract

The immune system protects the body against dangers that include pathogens, damage and cancer. Modern cancer therapies have sought to bolster immune responses against cancer using immunotherapy, which may include various forms of immune checkpoint therapy (ICT) in addition to methods of adoptive cell transfer (ACT), which is often associated with transfer of chimeric antigen receptor (CAR) T cells. Despite favorable outcomes in some patients and some cancers, as many as 60-80% of patients fail to benefit from ICT due to primary or adaptive resistance. This highlights the need for deeper understanding of how cancers suppress the immune system. Solid tumors, which make up approximately 90% of all cancers, are characterized by an immunosuppressive tumor microenvironment (TME). A hallmark of the TME is dysfunctional vascularization and impaired perfusion, which hinder effective drug delivery and promote hypoxia-induced metabolic reprograming in both cancer and immune cells. As the TME imposes intense metabolic stress through nutrient competition and lactate-driven acidification – both of which activates immunosuppressive pathways, targeting the TME itself may be beneficial in enhancing the efficacy of immunotherapy. Here we will briefly discuss the potential of targeting the metabolism of the TME as a means to promote normalized tumor vascularization and/or enhance anti-tumor immune responses.

## 1 Introduction

Cancer is a group of more than 100 diseases acquired by cellular defects, resulting in several hallmark features, such as uncontrolled cell growth, resistance to apoptosis, immune evasion, as well as metabolic dysregulation and metastasis (1). Approximately 90% of all cancers form tumors, which are composed of cancer cells, stromal cells and tissue-resident and infiltrating immune cells (2). The composition of the tumor, which includes all cellular and acellular factors is referred to as the tumor microenvironment (TME) (3–5). Cancers can arise in virtually all tissues of the body and is driven by a wide range of different inherited and acquired mutations, resulting in immense heterogeneity (6–8). At their core cancers are characterized by the loss of proliferative control, typically caused by the inactivation of tumor suppressor genes or the hyperactivation of oncogenes. Due to their role in regulating growth and cell cycle progression loss-of-function mutations in tumor suppressor genes results in loss of proliferative control, whereas activation of oncogenes promote uncontrolled growth (9–11). Most commonly, cancer is caused by mutations in genes encoding p53, PIK3CA, FAT4 and KRAS, with p53 mutations being observed in more than 50% of cancers (12, 13). Moreover, single-cell sequencing and spatial transcriptomics have further revealed that heterogeneity exists within the tumor itself (14–16).

The rapid rate of proliferation of cancer cells has been exploited therapeutically for decades. Chemotherapeutic drugs and radiotherapy preferentially target rapidly proliferating cells by either inducing DNA damage or by blocking central pathways involved in DNA replication (17). The rapid growth coupled with dysfunctional DNA repair boosts the accumulation of mutations, and thus accelerates the rate of cancer evolution. This makes the treatment of cancer especially challenging, as cancer cells not only develop resistance to chemotherapeutic drugs but can also adapt to harsh environmental conditions in the TME, such as hypoxia and nutrient deprivation, further enhancing their survival (18, 19). Although sequencing of cancer cells is increasingly used to predict treatment efficacy, primary and acquired therapy resistance still prevents efficient treatment of cancer in many patients (20). Moreover, as most cancer drugs work by targeting rapid proliferation, this also affects healthy cells with a high proliferation rate, often resulting in hair loss, gastrointestinal distress and immune suppression, among others (17). Together, this highlights the need for additional cancer targeting strategies. The ability to acquire resistance to treatment indicates that a single target is most likely insufficient to efficiently to adequately treat cancer patients. The identification of non-redundant pathways may result in a synergistic effect, thus requiring lower doses and potentially reducing adverse effects. This has been proven safe and effective in treatment of hypertension, and *in vivo* models suggest this also has the potential in cancer immunotherapies (21–23).

## 2 The immune system

The immune system is a complex, interactive network of defense and surveillance mechanisms, comprising physical barriers such as the skin and mucosal surfaces, as well as specialized lymphoid organs, immune cells and molecules. Together, these components work in concert to protect the host from non-self, including pathogens. The active immune system is divided into two core responses, namely the innate and the adaptive immune systems, which are fundamentally differentiated by their speed, precision, and capacity to resolve infections in addition to differ in their ability to develop immunological memory (24, 25). The innate immune system serves as the first line of defense, continuously surveilling the body for general signs of infection or damage by recognizing conserved molecular patterns - pathogen association molecular patterns (PAMPs) or damage-associated molecular patterns (DAMPs) (26). Upon detection of foreign or damaged self, cells of the innate immune system are rapidly activated and recruited from the circulation within minutes to hours, eliciting a response that can be sustained for several days (25). Although the innate immune system lacks immunological memory, professional antigen-presenting cells (APCs) can also induce activation of the adaptive immune system, composed of B and T lymphocytes that can maintain memory to specific pathogens lasting decades (25, 27–29). This task is mainly performed by type 1 macrophages (M1), dendritic cells (DCs), and B lymphocytes. APCs capture antigens, process them, and present resulting antigenic peptide fragments via their major histocompatibility complex class II (MHC II) molecules, expressed on their surface to activate adaptive immune cells in lymphoid tissues (25, 30, 31). While MHC II molecules are exclusively expressed by APCs, all nucleated cells express MHC class I (MHC I) molecules, which present endogenous antigenic peptides to enable immune surveillance and thereby the elimination of infected or abnormal cells (30, 31). For T cells, antigenic peptide-MHC complexes are recognized by the T cell antigen receptor (TCR), which can discriminate between self- and non-self-molecules with remarkable specificity (31–33). Forming an essential part of the TCR is the CD3 complex, functioning as an intracellular signaling hub that translates extracellular antigen recognition into downstream signaling events that drive T cell activation to differentiation, effector function, and clonal expansion (33).

T cells are broadly classified into two helper and cytotoxic T cells, designated CD4+ helper (Th) and CD8+ cytotoxic T cells (CTLs), respectively (24). CD4+ T cells recognize antigens presented on MHC II molecules and play a central role in regulating and coordinating immune responses. The CD4+ T cells can be broadly categorized into CD4+ effector cells, orTh cells, and regulatory T cells (Tregs). While CD4+ effector T cells are crucial for clearing infection and repairing tissue damage, Tregs are responsible for preventing excessive tissue damage and autoimmunity, striking a balance to maintain a functional and healthy immune environment (34–37). In contrast to CD4+ cells, CD8+ CTLs recognize antigens presented on MHC I molecules, and are responsible for directly eliminating target cells through the release of effector cytokines or cytotoxic granules (38). Although most effector T cells, including both CD4+ and CD8+ subsets, have a transient lifespan, a small fraction differentiates into memory T cells, thereby ensuring long-term immune surveillance against the same antigen (39).

## 3 Cancer immunotherapy

The immune system’s intrinsic role in defending against non-self has fueled the longstanding hypothesis that immune cells can recognize cancer cells as foreign and thereby be weaponized to eliminate them. Indeed, the antigenic composition of tumors differs significantly from that of their non-transformed tissue counterparts, a distinction largely driven by their genetic instability – a core hallmark of cancer (40–42). The concept of leveraging the immune system to combat cancer, now known as immunotherapy, was first systematically introduced in the 1890s by William B. Coley, who documented several cases of spontaneous remission after administering a cocktail of killed bacteria and their products to stimulate the immune system in patients with inoperable cancer (43). What began as a foundational discovery led to decades of rigorous research in cancer immunology, ultimately positioning immunotherapy as the fourth cornerstone of cancer treatment alongside surgery, radiotherapy and chemotherapy (44). Immunotherapy now encompasses a wide variety of treatments, including immune checkpoint therapy (ICT), which will be the focus of this review, adoptive cell transfer (ACT), such as chimeric antigen receptor (CAR) T cells, as well as engineered antibodies (reviewed extensively in (45)).

The concept of immunotherapy builds on the ability of controlling aberrant antigen-induced immune cell activation (46). T cell stimulation through the TCR/CD3 complex requires concurrent perturbation of co-receptors. Whereas the initial interaction of the TCR with the MHC molecule secure antigen-specificity, co-receptor signaling is essential for tuning the activation process. This tuning depends on a balance between activating and inhibitory signals induced by perturbation of cell surface receptors with distinct functions. This is exemplified by the interplay between CD28 and the cytotoxic T-lymphocyte-associated protein 4 (CTLA-4) molecules, which compete for binding of CD80 and CD86 expressed on APCs (33). Whereas stimulation of the CD28 molecule provides a positive signal, CTLA-4 engagement delivers an inhibitory signal, thereby modulating the magnitude of initial T cell activation (33, 47). Additionally, programmed cell death receptor 1 (PD-1), also referred to as CD279, is activated by PD ligand-1 or 2 (PD-L1/2) and downregulates T cell activity. In line with this, PD-1 stimulation has an important role in regulating immunological tolerance and is vital in preventing autoimmunity and collateral tissue damage (48–51). Additionally, inflammatory cytokines elicited by the inflammatory process are essential signaling molecules that drive a productive T cell response and facilitate memory formation by shaping the activation of specific differentiation pathways within the cell (52–54).

It is now well established that cancer cells can trigger immune responses. This phenomenon is demonstrated by the utilization of tumor-infiltrating lymphocyte (TIL) therapy, where T cells are isolated from resected tumors,expanded *ex vivo*, then transferred back to the patient (55, 56). However, they also evolve mechanisms enabling them to evade immune detection and destruction, making immune evasion a defining hallmark of cancer (57–59). During cancer evolution, these mechanisms are continuously sculpted under the selective pressure of immunosurveillance, a phenomenon known as immunoediting (59). In this process, as patrolling immune cells selectively eliminate highly immunogenic cancer cells, they simultaneously impose a selection pressure that favors the survival and expansion of rare subclones with immune-evasive traits. Over time, these subclones can adapt, proliferate, and ultimately become immune-resistant (59). Consequently, from the earliest stages of tumor development, the immune system edits tumor immunogenicity, leading to the emergence of an immunoedited tumor dominated by cancer cell variants that have successfully evaded immune control.

Immune evasion can occur through multiple, non-mutually exclusive mechanisms (reviewed in (59) and (60)), with loss of tumor antigen presentation being one of the most well-characterized, allowing cancer cells to effectively hide in plain sight. This can result from defects in the machinery responsible for antigen processing and presentation, such as downregulation or loss of MHC I molecules on the cell surface, a phenomenon observed in 40-90% of cancers which shields cancer cells from recognition and elimination by CTLs (61, 62). In blood cancers, this “invisibility cloak” has been successfully targeted through the development of monoclonal antibodies or CAR T cells, another form of ACT, that recognize and bind tumor-specific surface antigens independently of the MHC I receptor (63). However, these approaches have shown limited efficacy against solid tumors, which, as mentioned, account for approximately 90% of all cancers (2).

In addition to avoiding detection from the immune system, cancers can also suppress the effector functions of anti-tumorigenic immune cells. A key example is the co-option of the PD-1 pathway by cancer cells (48, 49, 51). In addition to activated T cells, PD-1 expression is also detected on B cells and natural killer (NK) cells. In all three cell types, PD-1 expression is normally decreased when an inflammatory response is resolved during acute antigen clearance (50, 64). However, in cases of persistent antigen exposure, such as cancer and chronic infections, PD-1 expression remains elevated, which contributes to T cell exhaustion (48, 51, 65, 66). The two PD-1 ligands, PD-L1 and PD-L2, exhibit distinct expression patterns. While PD-L2 is predominantly expressed on APCs in lymphoid tissues, PD-L1 is broadly expressed across hematopoietic (e.g., T cells, B cells, macrophages) and non-hematopoietic cells (e.g., endothelial cells) (51, 67). PD-L1 is upregulated by pro-inflammatory cytokines, particularly interferon-γ (IFN-γ), as a feedback mechanism to tune down immune activity (50, 68). However, in many solid tumors, PD-L1 expression is aberrantly elevated within the TME due to constitutive oncogenic signaling or as an adaptive response to inflammatory cues (51, 65).

To restore T cell activity in anti-tumor immune responses, antibodies targeting co-inhibitory receptors and their ligands have been developed (48, 51, 69, 70). These include, but are not limited to, immune checkpoint inhibitors targeting the CTLA-4 and PD-1 pathways, which have demonstrated clinical efficacy in certain cancers and patient subsets (70). However, as many as 60-80% of patients with solid tumors either fail to respond or experience only transient benefits from ICT, highlighting the diverse mechanisms tumors employ to evade the immune system (71, 72). In solid tumors, many of these mechanisms are driven by the TME, a complex and dynamic ecosystem encompassing cellular, physical and chemical components that are continuously restructured and manipulated throughout tumor progression. In addition to cancer cells, the cellular constituents of the TME include diverse non-malignant stromal cells along with tissue-resident and infiltrating immune cell populations that can be reprogrammed to support tumor growth (5, 73–75). In addition to cellular components, non-cellular components – such as the extracellular matrix (ECM), metabolites, soluble signaling molecules, and the surrounding hypoxic and acidic milieu – play important roles in tumor progression and therapy resistance (5, 73–75). Targeting the TME and its diverse components to enhance immunotherapy holds significant therapeutic promise, as many of its defining features are conserved across a range of tumor types (5, 75). In this review, we will provide a brief overview of key immunosuppressive hallmarks of the TME and discuss their potential as therapeutic targets to enhance immunotherapy in cancer, with a particular focus on ICT.

## 4 Cellular metabolism and metabolic reprogramming

Over the last decades it has become clear that extracellular metabolites are crucial for optimal T cell activation. Nutrients and their metabolites exhibit significant interplay with the three core activation signals while also independently influencing the functional polarization of T cells. Consequently, metabolic inputs have been proposed as a novel dimension necessary for licensing the T cell immune response (reviewed in (76) and (77)). The connection between cellular function and metabolic phenotype in health and disease is therefore becoming increasingly evident across multiple fields, including immunology, cancer, and tumor angiogenesis (41, 52, 78–83). While energy and biomass production remain critical, recent insights explain how metabolism affects diverse processes such as activation, proliferation, migration and differentiation.

**Figure 1:**
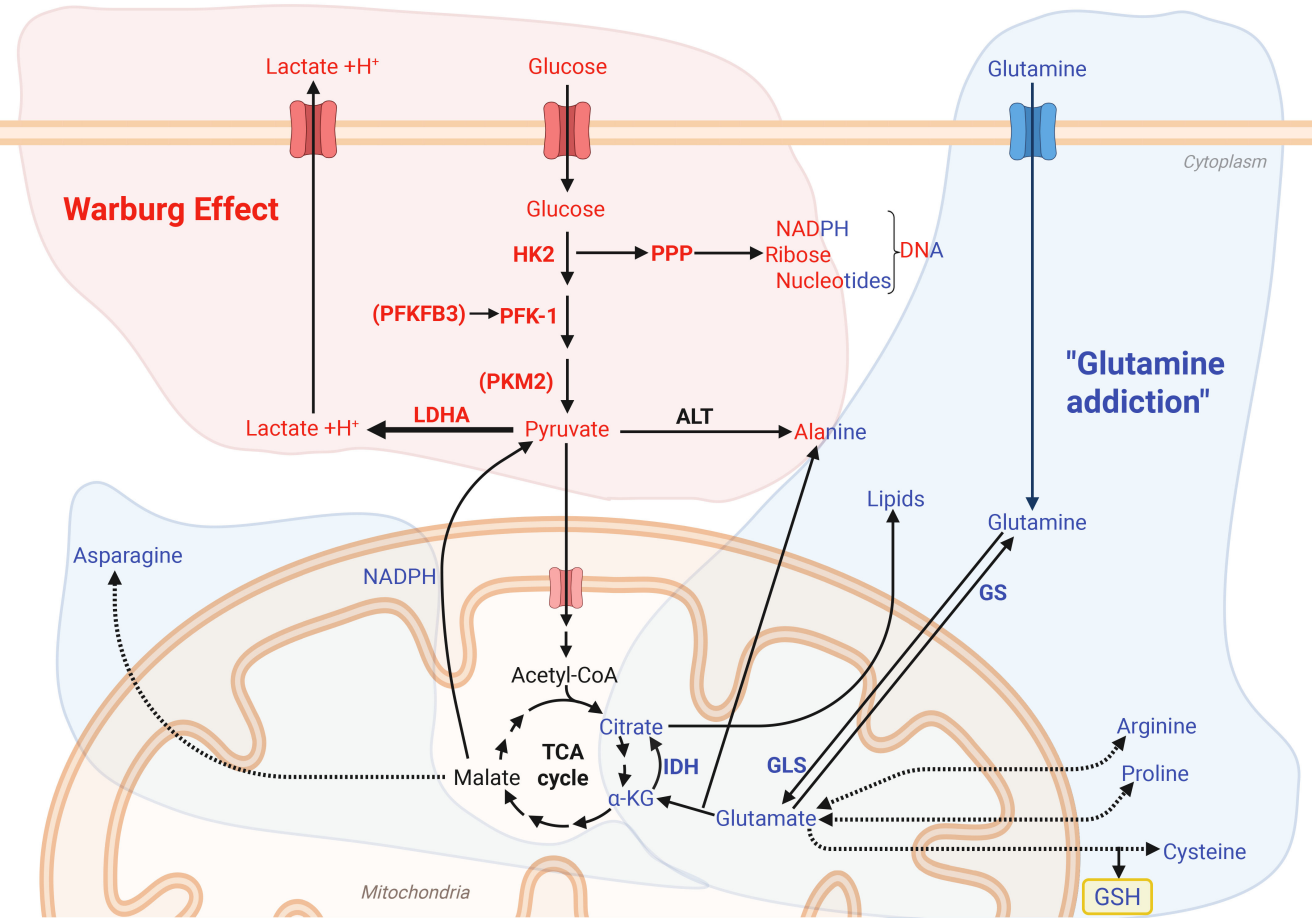
The Warburg effect and glutamine metabolism drives energetic and biosynthetic pathways. The Warburg effect (84, 85) metabolizes glucose into lactate despite the presence of oxygen, as well as providing important glycolytic metabolites which support redox homeostasis through the pentose phosphate pathway (PPP) and the carbohydrate backbones for DNA and RNA synthesis. Glutamine addiction (blue) describes the reliance on glutamine to provide precursor for amino acid synthesis, fueling the TCA cycle for regeneration of NADPH and generation of reduced glutathione (GSH). Figure was made using BioRender.

Although metabolic reprograming has only recently emerged as important in these processes, it has been known in cancer cells for close to a century. As early as in the 1920’s, Otto Warburg discovered that cancer cells, despite the presence of oxygen, preferentially rely on glycolysis and lactate production rather than mitochondrial respiration, a metabolic reprogramming now known as “The Warburg effect” (**Figure 1**) (84, 85). Cancer cells frequently upregulate glucose transporters and key enzymes in the glycolytic pathway, including Glucose transporter type 1 (GLUT1), Hexokinase 2 (HK2), 6-phosphofructokinase 2/fructose 2,6-bisphosphatase 3 (PFKFB3), pyruvate kinase muscle form 2 (PKM2) and lactate dehydrogenase A (LDHA) (86). Together, these proteins facilitate a rapid turnover of glucose and a subsequent increase in lactate production. Although this metabolic phenotype was originally believed to be caused by a mitochondrial defect in cancer cells, it is now recognized that the mitochondria remain functional and possess remarkable metabolic plasticity. This flexibility enables them to dynamically utilize a wide range of substrates, including glutamine and fatty acids to fuel diverse cellular processes (**Figure 1**) (87–89).

This metabolic plasticity may also provide resistance to metabolic inhibitors, as demonstrated by Boudreau et al. (90), where the glycolytic pancreatic cancer cell line MIA PaCa-2 adopted an oxidative phenotype after long-term exposure to an inhibitor of lactate production. In line with this, glutamine reliance, a phenomenon sometimes referred to as “glutamine addiction”, is observed in several cancer cell lines (83, 87, 88, 91, 92). Although most cells are capable of synthesizing glutamine, rapidly proliferating cells rely on additional extracellular sources, so it is sometimes referred to as a conditionally essential amino acid (88, 91, 93). Interestingly, glutamine deprivation has been shown to reduce the rate of glycolysis by regulating both the expression and activity of key glycolytic enzymes (94, 95). Furthermore, the expression of the enzyme glutaminase 1 (GLS1), which is responsible for the deamidation of glutamine to glutamate, is upregulated in many cancer cell lines and correlates with decreased survival in patients (96). Indeed, Reinfeld et al. (97) showed that myeloid cells and T cells have a higher capacity for glucose uptake than cancer cells, while cancer cells have a higher capacity for glutamine uptake. The enzymes isocitrate dehydrogenase (IDH) 1 and 2 are also important in the TCA cycle, as they catalyze the formation of α-ketoglutarate from isocitrate (98). This supports redox homeostasis and lipid synthesis, along with providing α-ketoglutarate which can regulate epigenetics or be used as a backbone for glutamate and glutamine synthesis (98–100). In cancers, mutations in IDH1 or IDH2 can result in the formation of D-2-hydroxyglutarate (2-HG) (98). Some cancer cells also rely on fatty acid oxidation, a trait which is associated with upregulated expression of the mitochondrial fatty acid transporter carnitine palmitoyl transferase 1 (CPT1) in certain tumors (89).

**Figure 2:**
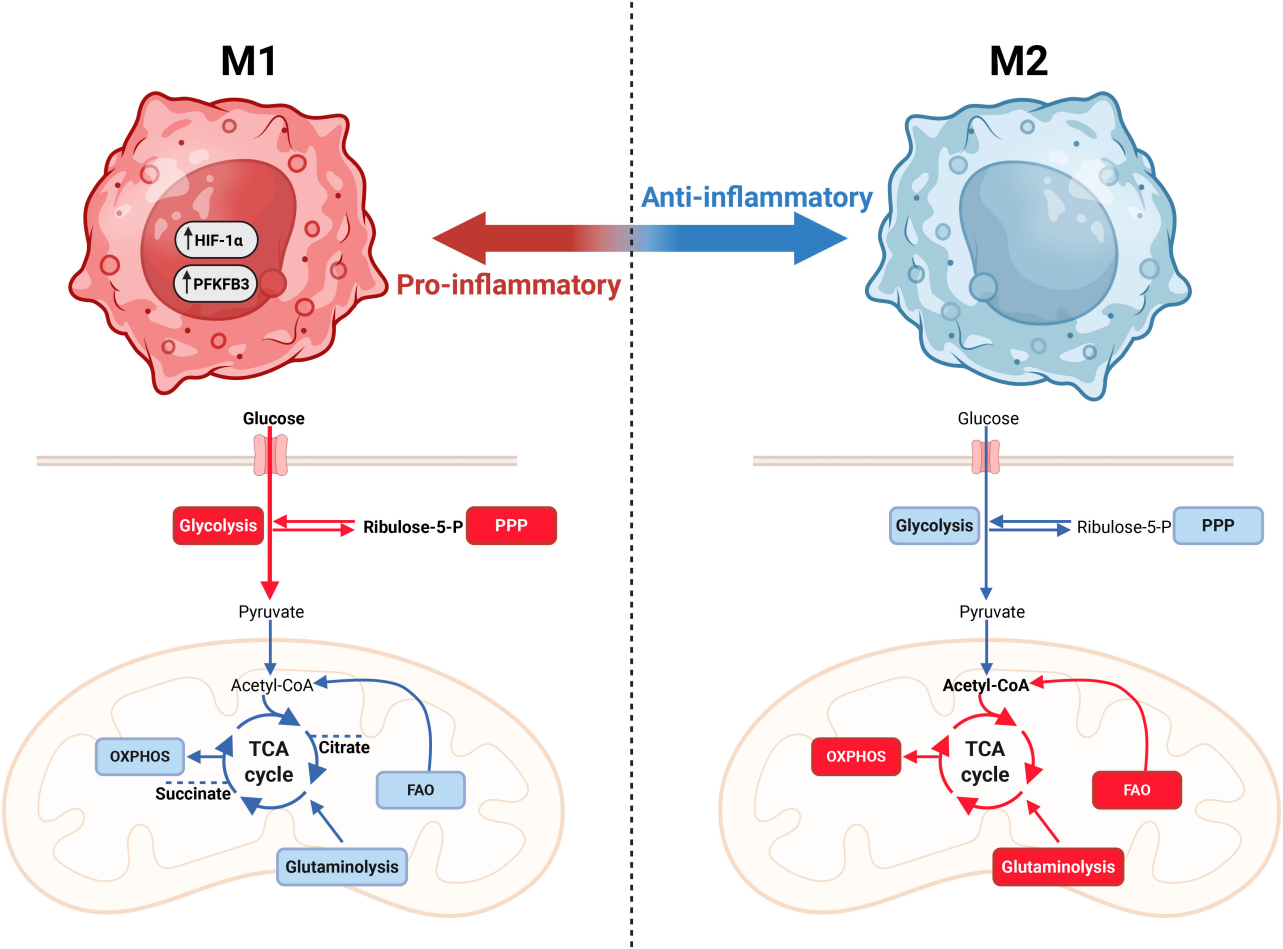
Macrophage metabolism is closely linked with effector function. Macrophages can polarize into pro-inflammatory (M1-like) and anti-inflammatory (M2-like) phenotypes. M1 macrophages are characterized by a high reliance on glycolysis and a less oxidative phenotype. Additionally, M1 macrophages utilize the TCA cycle to produce citrate and succinate to drive stabilization of HIF-1α to aid pro-inflammatory effector functions. The anti-inflammatory, wound-healing M2-like macrophages are less glycolytic and more oxidative, utilizing glutamine and fatty acid oxidation (FAO). Figure was made using BioRender.

Metabolic plasticity and reprogramming are important for both cancer and immune cells. An increasing body of evidence demonstrates that, in some contexts, healthy cells - including immune cells and endothelial cells - can adopt similar metabolic phenotypes (101–103). Indeed, immune cells, including macrophages and T cells, undergo metabolic reprogramming upon activation (81). Macrophages, which are traditionally classified into pro-inflammatory M1 or anti-inflammatory, wound-healing M2 subtypes, adapt to a glycolytic and oxidative metabolic program, respectively (**Figure 2**) (104–109). Similar to cancer cells, M1 macrophages upregulate PFKFB3 to boost the rate of glycolysis (104). In line with this, deletion or inhibition of PFKFB3 in macrophages is shown to result in reduced secretion of pro-inflammatory cytokines, including IL-1β, IL-6 and tumor necrosis factor following stimulation with lipopolysaccharide. Moreover, mice with a myeloid-specific PFKFB3 deficiency exhibit increased survival in a murine sepsis model, as well as increased lymphangiogenesis following myocardial infarction (110, 111). Additionally, the tricarboxylic acid (TCA) cycle is used by M1 macrophages to produce succinate, which stabilizes hypoxia-inducible factor 1 α (HIF-1α), and citrate, which serves as a precursor for fatty acid synthesis and the antimicrobial metabolite itaconate (112, 113). In contrast, M2 macrophages utilize glutamine and fatty acids as substrates for the TCA cycle, fueling adenosine triphosphate (ATP) production through oxidative phosphorylation (114). Additionally, they metabolize tryptophane via the enzyme Indoleamine 2,3-dioxygenase (IDO) to generate the anti-inflammatory metabolite kynurenine (115, 116). IDO1 activity suppresses T-cell activity by depleting tryptophan, which is required for Th1 and CD8+ T-cell-mediated immunity, while kynurenine binds to the aryl hydrocarbon receptor (AHR), which directly activates Treg differentiation and activity, resulting in reduced anti-tumor responses (116–118).

**Figure 3:**
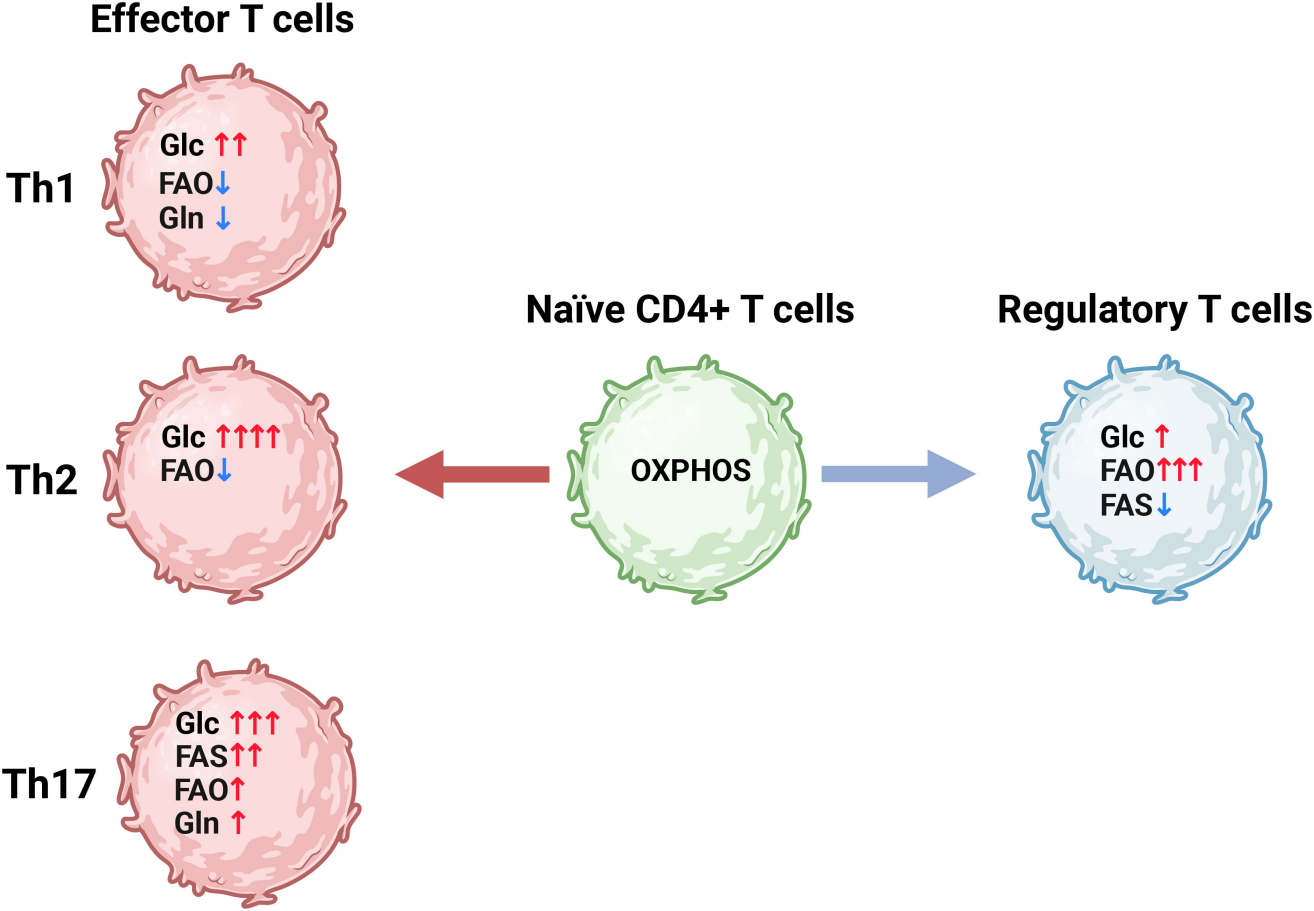
CD4+ T cell differentiation is correlated with distinct metabolic programs. Naïve CD4+ T cells (green) have a relatively low metabolic rate driven mainly by oxidative phosphorylation (OXPHOS). Upon activation the naïve CD4+ T cells differentiate into various subsets of effector CD4+ T cells (Th) (red) which utilize distinct metabolic programs characterized by differential reliance on glycolysis (Glc) glutamine metabolism (Gln) fatty acid oxidation (FAO) and fatty acid synthesis (FAS). Alternatively, CD4+ T cells can differentiate into regulatory T cells (Tregs), which are less glycolytic and rely more on FAO to fuel ATP production compared to the effector CD4+ T cells. Figure was made using BioRender.

Metabolic reprogramming is also important for T cells, with different T cell subsets adopting distinct metabolic adaptations (**Figure 3**) (103, 119, 120). In their naïve state, T cells primarily rely on oxidative phosphorylation to produce ATP, maintaining a relatively low metabolic rate prior to activation. However, upon activation through perturbations of the TCR/CD3 complex in conjunction with CD28, their metabolic rate rapidly increases (121, 122). This process is aided by the presence of abundant mRNA encoding key glycolytic enzymes, particularly HK2, along with the availability of idle ribosomes, enabling the rapid production of proteins upon upregulation (121). Additionally, activation of Akt, also known as protein kinase B, quickly upregulates the cytosolic localization of GLUT1 required to increase glucose uptake (123). Chang et al. (124) demonstrated the metabolic plasticity of T cells by replacing glucose with galactose. Despite their inability to metabolize galactose through glycolysis, T-cell proliferation is reportedly unaffected by replacing glucose with galactose, however glucose deprivation resulted in decreased production of the cytokine interferon-γ (IFN-γ), as idle glyceraldehyde 3-phosphate dehydrogenase (GAPDH) bound the IFN-γ mRNA and prevented its translation (124) In addition to glucose, T cells also require glutamine to become fully activated, and glutamine deprivation or inhibition of GLS1 reduces both proliferation and cytokine secretion in CD4+ T cells (125–128). In fact, we recently demonstrated that, similar to cancer cells, glutamine deprivation in CD4+ T cells also regulate glycolysis (129). Furthermore, CD4+ T cell subsets also adapt to distinct metabolic profiles (**Figure 3**), as previously reviewed in detail (130)), highlighting the close relationship between metabolism and functionality in T cells.

**Figure 4:**
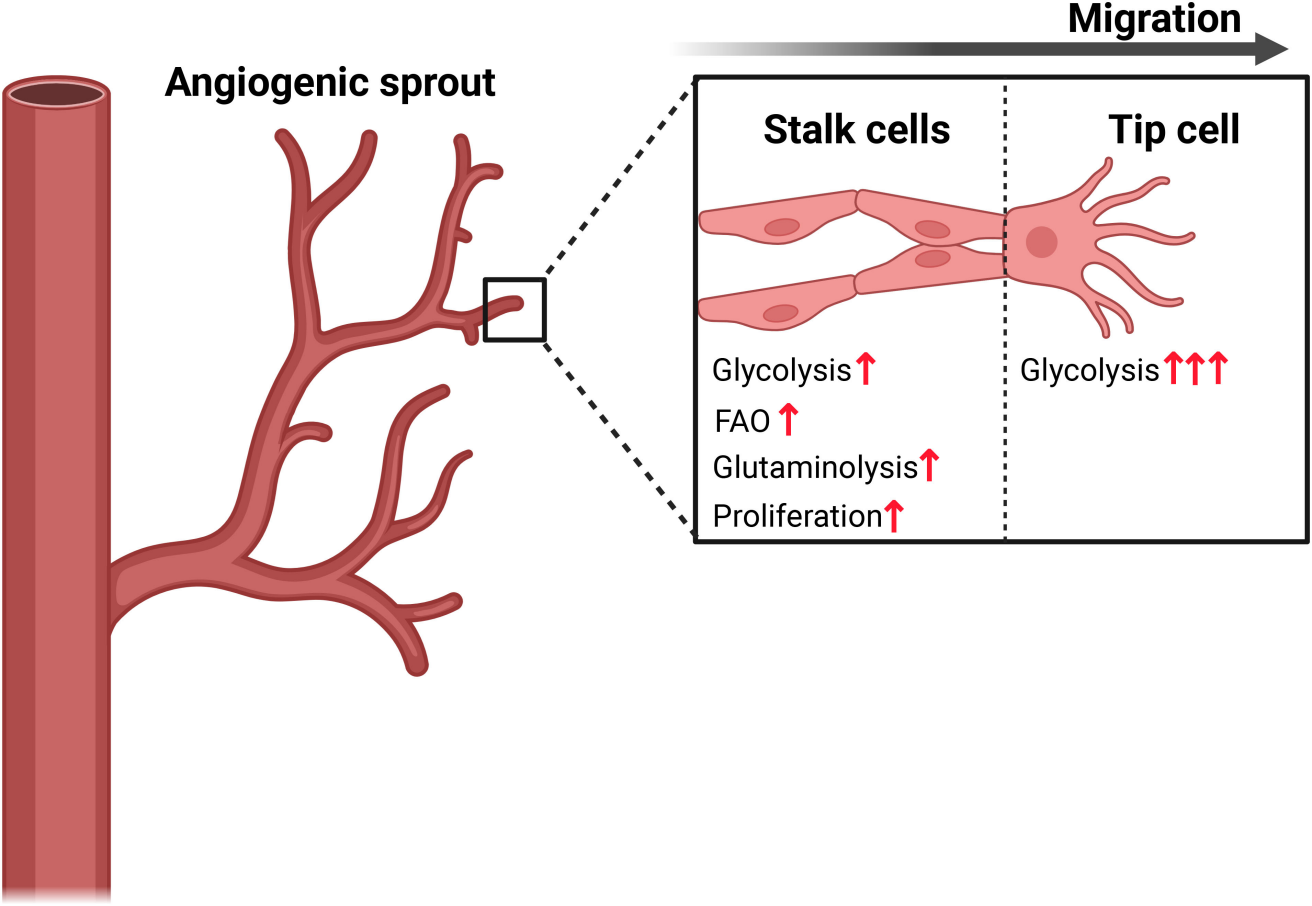
Endothelial cell metabolism drives migration and proliferation in vessel sprouting. The highly migratory endothelial tip cell is characterized by a higher rate of glycolysis driven by expression of PFKFB3, while the stalk cells rely on a combination of glycolysis, fatty acid oxidation (FAO) and glutaminolysis to provide building blocks required for rapid biomass proliferation. Figure was made using BioRender.

Metabolism in endothelial cells has also been extensively studied due to their critical role in angiogenesis. Importantly, deciphering metabolism in these cells has led to the identification of therapeutic targets with the potential to regulate angiogenesis (reviewed in (131)). It is known that glycolysis may account for up to 85% of the ATP production in endothelial cells despite sufficient levels of circulating oxygen (102). Notably, the rate of glycolysis can be upregulated in response to pro-angiogenic and inflammatory factors (102, 132–135). In fact, upregulation of glycolysis through increased expression of PFKFB3 is a strong driver of tip cell formation in vessel sprouting (102, 135). However, the proliferating tip and stalk cells, which are the building blocks of sprouting angiogenesis, rely not only on glycolysis, but also on fatty acid oxidation to produce dNTPs for DNA synthesis, and on glutamine metabolism to fuel the TCA cycle, thereby driving vessel propagation (**Figure 4**) (93, 101, 136).

Despite the differences in metabolic profiles, most of the regulatory mechanisms are shared and conserved across cell types. Among these are mammalian target of rapamycin complex 1 and 2 (mTORC 1 and 2), HIF-1α and c-MYC (137–140). mTOR is a serine/threonine kinase that functions as part of two distinct complexes: mTORC1 and mTORC2. mTORC1 is an important regulator of anabolic metabolism, including protein and lipid synthesis, while also supporting catabolic processes by enhancing glycolysis through stabilization of HIF-1α and inducing enzymes responsible for glutaminolysis via the transcriptional activity of c-MYC (141). mTORC2 is associated with cell survival and fine-tuning of metabolic activity. It promotes fatty acid oxidation by regulating the transcription factor Forkhead box 01 (FOXO1) and enhance glycolysis through activation of Akt (142).

Stabilization of HIF-1α induces glycolytic metabolism through upregulating expression of GLUT1, HK, PFKFB3, PKM2, LDHA and Monocarboxylate transporter 4 (MCT4) (104, 143–146). Although HIF-1α is primarily stabilized by the absence of oxygen, several mechanisms can also promote its stabilization in the presence of oxygen, including the regulation by mTORC1 (141, 142). In cancer, HIF-1α stabilization is associated with a dismal prognosis for patients (147, 148). However, it also plays an important role in the functional activation of immune cells, highlighting its dual role in both promoting cancer cell growth and supporting anti-tumor immunity [reviewed in detail (149)].

## 5 The tumor microenvironment drives immune suppression

**Figure 5:**
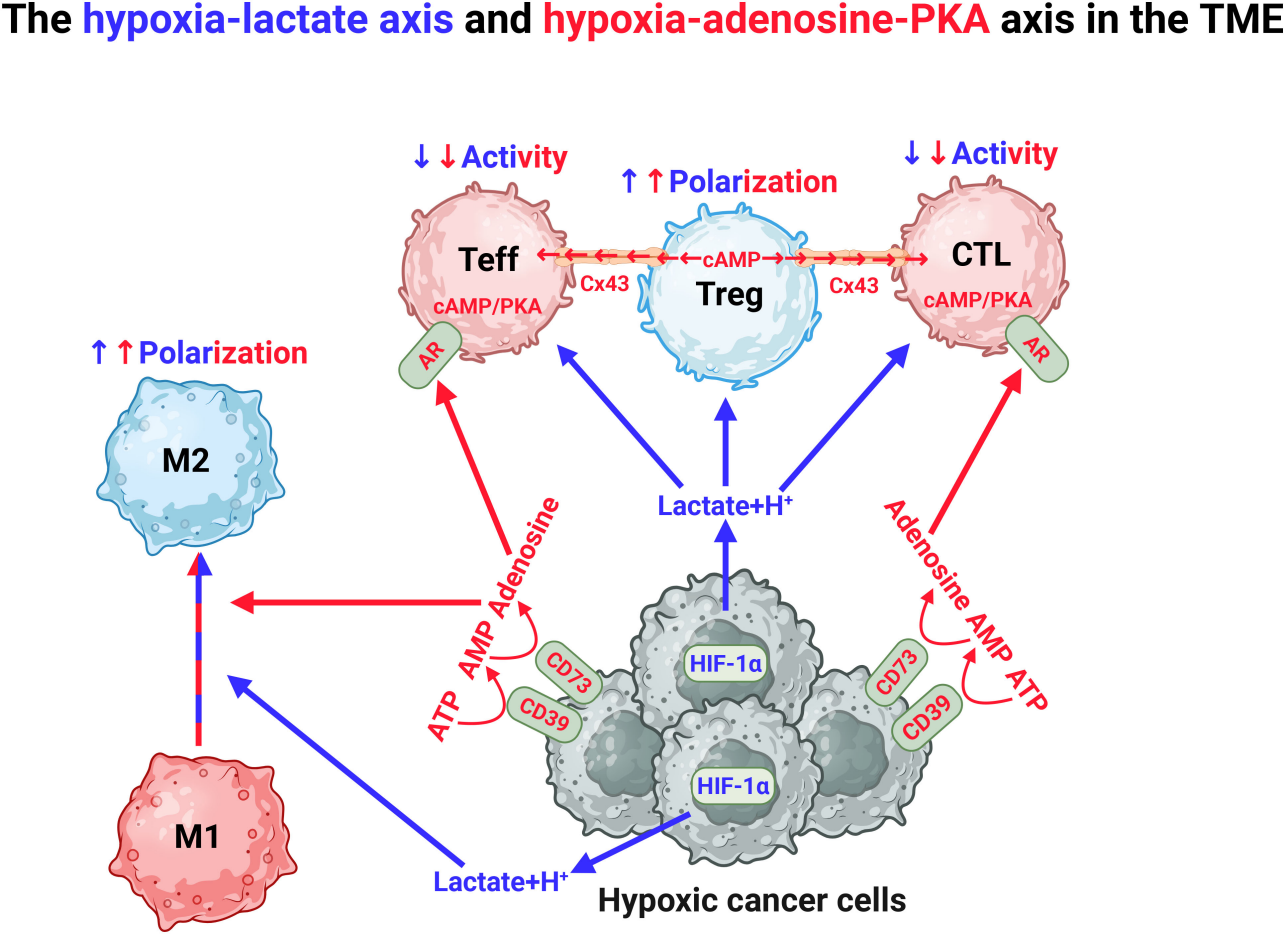
Hypoxia in the TME drives immunosuppression through the hypoxia-lactate axis and the hypoxia-adenosine-PKA axis. The TME is characterized by the presence of hypoxia. This drives the production of lactate, which induces polarization of anti-inflammatory M2 macrophages and Tregs. Hypoxia also induces expression of CD39 and CD73 and stepwise production of adenosine, which stimulates adenosine receptors (AR) that produce endogenous cAMP in CD4+ effector cells (Teff) and cytotoxic lymphocytes (CTL). Cyclic AMP is also produced by Tregs which transfer this to Teffs and CTLs through the junction protein connexin 43 (Cx43). In both situations, endogenous cAMP will induce PKA activation leading to inhibition of proinflammatory function of Teff and CTL. Figure was made using BioRender.

The TME is generally poorly vascularized, with dysfunctional and leaky blood vessels resulting in decreased availability of nutrients, hypoxia and acidification that collectively contribute to immune suppression through several mechanisms (**Figure 5**). Whereas hypoxia is associated with increasing tumor mass, acidification is associated with reprogrammed cancer cells producing lactate. Tumor hypoxia induces stabilization of HIF-1α which, as mentioned, drives glycolysis and is hence responsible for the lactate production (4, 5, 74, 75, 97, 150). Lactate accumulation is known to restrict T-cell proliferation by disrupting the redox homeostasis and inhibiting GAPDH activity (151). Interestingly, T cells produce acidic niches within lymph nodes to restrict their own effector functions to avoid hyperactivation, demonstrating the physiological importance of lactate (152). In addition to acidification, lactate is known to induce histone modification referred to as lactylation, which is associated with enhanced polarization of M2 cells, suppressed T cell effector functions and increased Treg differentiation, thereby supporting an anti-immune and pro-tumorigenic phenotype (153–155). In line with this, reduced lactylation has been associated with a favorable outcome in patients with solid tumors (153, 156). In addition to regulating glycolysis, HIF-1α stabilization can also act immunosuppressive by inducing the expression of PD-L1 in DCs, macrophages and myeloid derived suppressor cells (149). The lactate-induced acidification has also been shown to reduce the efficacy of immune checkpoint inhibition by influencing the binding properties of antibodies targeting PD-L1 (157). The hypoxia-lactate axis also contributes to the formation of dysfunctional blood vessels, together favoring tumor metastasis and repression of immune cell infiltration (158, 159).

Tumor hypoxia also induces expression of CD39 and CD73, which together catalyzes the formation of adenosine from adenosine monophosphate (AMP) and ATP. Adenosine exerts its effects through ligation of the 4 subtypes of purinergic adenosine receptors (A1, A2A, A2B, and A3) (160, 161). The ARs differ in their affinity for adenosine, but are all coupled to various cellular signaling pathways through G-protein coupled receptors (GPCRs). To this end, A2AR and A2BR are upregulated in response to hypoxia and anergic signaling in T cells and induce immune suppression by activating adenylate cyclases (ACs), that initiate the synthesis of intracellular cyclic AMP (cAMP), which in turn activates the cAMP-dependent protein kinase A (PKA) (161). PKA is a holoenzyme consisting of a regulatory (R) subunit dimer and two catalytic (C) subunits. While the R subunits are encoded by four separate genes (PRKAR1A and -B, PRKAR2A and -B), the catalytic subunits are primarily encoded by two major genes, PRKACA and PRKACB, which give rise to several tissue- and cell-specific splice variants, including immune cell-specific Cβ2 (162, 163). In addition to Cβ2, the splice variants Cα1 and Cβ1 are expressed in immune cells. The prevailing dogma is that PKA activation suppresses both early and late phases of T cell activation, including proliferation and clonal expansion, the latter mediated by downregulation of IL-2 production (164, 165). In line with its inhibitory role, PKA also promotes differentiation into Tregs and Th2, which favors the tumor, while repressing effector functions of Th1, Th17 and CD8+ T cells (166, 167). Additionally, Tregs can directly induce PKA activation by transporting cAMP into target T cells through the gap-junction protein connexin 43 (Cx43) (168). As a result, in the TME, PKA activation drives T cell exhaustion, in conjunction with upregulation of PD-1 and CTLA-4 expression (72). Recently, we showed that deletion of the immune-specific PKA Cβ2 significantly suppressed tumor growth and enhanced survival in a murine metastatic cancer model (169). This was associated with increased infiltration of pro-inflammatory Th1, Th9 and Th17 cells into the tumors (169). Moreover, in 2020, Na et al. demonstrated that knockout of PKA Cβ in macrophages prevents M2 polarization and that liposomal delivery of PKA inhibitors to tumor-infiltrating macrophages enhances the therapeutic efficacy of anti-CTLA-4 antibodies, effectively counteracting breast cancer tumor growth and metastatic potential in mice (170). Together this suggest that Cβ may convey signals supporting a proinflammatory phenotype. In support of this, mice that are ablated for Cβ2 are prone to develop autoimmunity, a phenotype also reflected in upregulation of proinflammatory immune cells (171).

In addition to the hypoxia-lactate-adenosine axis, some cancer mutations result in metabolic phenotypes that contribute to an immunosuppressive TME. Cancer cells with IDH1 or IDH2 mutations cause accumulation of 2-HG, which supports the cancer cells by maintaining a stem-like phenotype, while suppressing T-cell- and macrophage-mediated immune activity (172). 2-HG is taken up by CD8+ T cells, where it destabilizes HIF-1α and acts as an inhibitor of LDH, resulting in reduced chemotaxis, cytotoxic activity and production of IFN-γ (172–174).

## 6 Targeting the tumor microenvironment

As the TME exerts broad immunosuppressive characteristics, targeting the TME also offers the potential for new therapies. As mentioned, the hypoxia-lactate axis and the hypoxia-adenosine-PKA axis are known to inhibit the immune system. Thus, targeting hypoxia directly by hyperbaric oxygen (HBO) treatment has been proposed (175). Although this approach is reported to enhance immune activity and reduce growth of pulmonary tumors in a mouse model (176) a recent meta-analysis found overall weak evidence that HBO treatment alone improves long-term survival (176) and its use in humans remains limited due to the lack of high-quality studies (177). Although HBO treatment might not be effective as a cancer treatment, targeting the downstream effects of hypoxia on metabolism, PKA activity and angiogenesis may still have therapeutic benefits. Moreover, targeting IDH1 and IDH2 have been explored. This is due to the fact that the cancer-specific mutation of IDH has potential to be targeted without affecting the non-mutated IDH isoforms in healthy cells (178).

### 6.1 Targeting tumor vascularization

The hypoxia-angiogenesis-axis has also been explored as a therapeutic strategy, which has led to the approval of monoclonal antibodies targeting VEGF for use in some cancers, including renal and colorectal cancer (179, 180). When used in combination with chemotherapy, VEGF blockade has been shown to increase progression free survival in many types of cancer (181). The combination of anti-angiogenic drugs with ICT has been proposed as a strategy to increase immune cell infiltration and improve therapeutic efficacy (182). It is also hypothesized that rather than blocking angiogenesis, it might be more beneficial to normalize the tumor vasculature to enhance vascular integrity and improve tumor perfusion (3). However, although VEGF is the main driver of angiogenesis, it is well established that additional, not yet fully understood mechanisms can also contribute to this process (131). These VEGF-independent pathways may aid resistance to VEGF-targeted therapies, highlighting the need to identify and target alternative pro-angiogenic signals.

The Notch pathway, which is known for regulating angiogenic and inflammatory pathways in endothelial cells, is of interest in targeting pathological angiogenesis [reviewed in ref (183)]. Notch signaling is induced when one of the four Notch receptors (1–4) is activated by binding to a ligand from the Delta-like (DLL1, DLL4) or Jagged (Jag1, Jag2) families (183). The Notch pathway is traditionally viewed as anti-angiogenic, and its inhibition leads to increased angiogenesis (102). However, we recently demonstrated that blocking Jag1 resulted in an upregulation of DLL4, which we and others, have reported reduces expression of VEGFR2 (184–186). Jag1 blockade was further associated with a reduction in M2-like macrophages, which might be also enhance the immune function in the TME as well (185).

Targeting endothelial cell metabolism has been proposed as a strategy to bypass resistance mechanisms, an approach that Treps et al. (131, 187) describe as “targeting the engine of angiogenesis”. In accordance with this, targeting endothelial glycolysis, glutamine metabolism and fatty acid metabolism have been explored as therapeutic strategies for pathological angiogenesis (187).

Inhibition of endothelial glycolysis through PFKFB3 blockade has been shown to reduce pathological angiogenesis in several disease models, including tumor neovascularization (132, 135, 188). Both pharmacological inhibition and partial deletion of endothelial PFKFB3 reduced tumor vascularization and metastasis, while also increasing vessel stability, which aided drug delivery and enhanced the effect of chemotherapy in a murine liver cancer model (132). Additionally, it was demonstrated that endothelial PFKFB3 and lactate enhanced polarization of M2-like macrophages in a murine ischemia model, suggesting a potential role in modulating immune responses in cancer as well (189). Lactate accumulation also directly influences angiogenesis by stabilizing HIF-1α and promoting vascularization, while simultaneously reducing vessel integrity through the activation of inflammatory pathways. Moreover, prolonged exposure to lactate drives endothelial dysfunction in the TME (74, 190, 191). Although inhibition of PFKFB3 has also been demonstrated to reduce cancer cell proliferation, the concentrations of the PFKFB3 inhibitor 3-(pyridin-3-yl)-1-(pyridin-4-yl)prop-2-en-1-one (3PO) required to achieve this effect were shown to simultaneously reduce vessel integrity and facilitate metastasis (191–193). Moreover, as there are numerous reports of off-target effects associated with 3PO, further studies are needed to determine the safety and feasibility of targeting PFKFB3 in the context of tumor vascularization (133, 194–196).

Glutamine metabolism also presents a promising target in tumor vascularization, as its importance is shared between the endothelium and the tumor (83, 93, 96, 136, 197). GLS1 inhibition has been shown to be highly effective in reducing endothelial cell proliferation as it provides an important precursor for α-ketoglutarate and the amino acid asparagine (93, 136). This approach holds potential for a synergistic effect because, as mentioned, cancer cells rely on glutamine to fuel the TCA cycle (96). Although GLS1 inhibition also reduces proliferation and cytokine secretion from CD4+ T cells, this may be circumvented by using the GLS1 inhibitor telaglenalstat (CB839). Although CB839 has been shown to have an inhibitory capacity (IC50) in the nanomolar range in sensitive cancers, it appears to inhibit proliferation without inducing apoptosis in endothelial cells in the micromolar range, while we have showed that proliferation of CD4+ T cells is not significantly reduced by concentrations up to 5 micromolar (125, 126). Moreover, CB839 also appears to induce an M1-like phenotype in macrophages, which may further enhance the anti-cancer response of the immune system (198). Additionally, glutamine deprivation or GLS1 inhibition represses glycolysis and lactate production in several cancer cells through upregulation of thioredoxin interacting protein (TXNIP) and phosphorylation of PFKFB3, potentially reducing lactate-induced differentiation of M2 macrophages (94, 95). Further studies will be needed to assess the potential synergistic effects of GLS1 inhibition and immunotherapy.

Fatty acid oxidation is another important pathway in proliferating endothelial cells. As demonstrated by Schoors et al. (101), endothelial cells use fatty acids to produce nucleotides for DNA synthesis, and inhibition of the mitochondrial fatty acid transporter CPT1A reduces proliferation. CPT1A is upregulated in certain cancers and is associated with resistance to induction of apoptosis (199, 200). Moreover, CPT1A is important for driving Treg differentiation (199, 200). CPT1A also seems to drive an anti-inflammatory phenotype, as deletion of CPT1A resulted in increased lung damage in a murine LPS-induced sepsis model (201) while inducing CPT1A expression in the RAW264.7 macrophage cell line reduced expression of iNOS and impaired phagocytotic capacity (202). In line with this, targeting CPT1A has been shown to enhance the effect of PD-1 blockade in a murine lung cancer model (199).

Adenosine, produced in response to tumor hypoxia, is another key driver of angiogenesis, promoting HIF-1α stabilization and VEGF production through ligation of the A2AR (203, 204). Stimulation of A2AR enhances glycolysis in endothelial cells, making the hypoxia-adenosine axis a promising target for modulating both the immunosuppressive and the pro-angiogenic features of the TME (204). The PKA pathway also plays a crucial role in angiogenesis by regulating endothelial cell proliferation, migration and modulating VEGF signaling (203, 205). Another cAMP effector, exchange protein directly activated by cAMP (Epac), contributes to the regulation of angiogenesis by inhibiting γ-secretase, an enzyme required for the intracellular cleavage Notch and thereby activation of the Notch pathway. In line with this, inhibition of Epac has been shown to reduce pathological angiogenesis by enhancing Notch activation and suppressing VEGF signaling (206).

### 6.2 Targeting lactate production in the TME

Due to the central role of lactate in the TME, targeting its production and transport have been proposed as potential therapeutic strategies (207). Given the extensive study of glycolysis and lactate production (74, 82, 190, 208), a myriad of inhibitors has been developed against key glycolytic enzymes, including LDH, HK, PFKFB3, and PKM2 in addition to the lactate transporters MCT1 and MCT4 (82, 195, 209–211).

When the glucose analog 2-deoxy-D-glucose (2-DG) inhibits HK, glycolysis is completely blocked. Although 2-DG has been shown to reduce proliferation in various cancer cells, its therapeutic potential is limited due to low specificity and hence off-target effects and toxicity – including immune suppression and gastrointestinal distress – together highlighting the need for more precise approaches (212–214).

PFKFB3, which is upregulated in many cancers, has been proposed as a more cancer-specific therapeutic target. This has resulted in extensive research into developing PFKFB3 inhibitors [reviewed in detail in (215)]. Although PFKFB3 is not directly involved in glycolysis, PFKFB3 inhibition reduces lactate production and proliferation in several cancer cell lines (209, 216). However, PFKFB3 expression is also important in immune cells, including M1 macrophages and effector T cells (109, 217). In line with this, treatment with the PFKFB3 inhibitor 3PO alleviated inflammation and reduced mortality in murine sepsis models (110). Paradoxically, PFKFB3 expression is reported to correlate with pro-invasive and pro-inflammatory activity in rheumatoid arthritis patients (218, 219), while selective inhibition of endothelial PFKFB3 reduces polarization of M2 macrophages (189). These contradictory findings stress the need for more research to explore how PFKFB3 inhibition may affect the cancer-immune interaction.

Direct targeting of LDH prevents the production of lactate and results in accumulation of pyruvate and NADH (208). This has been shown to induce oxidative stress and inhibit tumor progression in glycolytic cancers. However, cancer cells can circumvent this effect by rewiring their metabolism towards an oxidative phenotype, indicating that targeting LDH alone is insufficient (90, 220). Still, as reducing lactate levels in the TME might enhance the anti-tumor activity of the immune system, targeting LDHA still holds therapeutic potential. Notably, Renner et al. and Babl et al. (210, 221) showed that blocking lactate secretion enhanced the efficacy of anti-PD-L1 treatment by alleviating the immunosuppressive effects of lactate on T cells. Moreover, it was recently shown that the lactate-induced acidification of the TME also negatively affects the interaction between PD-L1 and anti-PD-L1 antibodies (157). Pilon-Thomas et al. (222) demonstrated that buffering the pH within the TME using sodium bicarbonate enhanced the anti-tumor activity of TILs, indicating that targeting the hypoxia-lactate axis may enhance the efficacy of several forms of immunotherapy.

### 6.3 Targeting the hypoxia-adenosine-PKA axis

As mentioned, the hypoxia in the TME induces expression of the ectonucleotidases CD39 and CD73, resulting in conversion of ATP to adenosine, which facilitates immune suppression through binding to the adenosine receptors A2AR and A2BR (160, 161). In turn, activation of A2AR and A2BR leads to AC-induced cAMP production and activation of PKA and Epac (223). Endogenous cAMP production is also induced by numerous other receptors, including β-adrenergic receptors, dopamine receptors, and prostaglandin receptors (PGER) such as PGE2R (223). Activation of PKA is known to suppress the nuclear factor of κ-light chain of activated B cells (NFκB) and STAT1 pathways in macrophages, resulting in a shift towards M2 macrophage polarization (72, 170, 224). In T cells, PKA phosphorylates C-terminal Src kinase (Csk), that phosphorylates lymphocyte-specific protein tyrosine kinase (Lck) on tyrosine 505, preventing downstream activation of T-cell signaling, including the NFκB and NFAT pathways (164, 225). This further results in decreased differentiation of CD4+ T cells to Th1 and Th17 cells, while increasing differentiation of Tregs (166, 167). The PKA pathway thus serves as an immune checkpoint, which offers potential as a therapeutic target.

Targeting the adenosine axis through the CD73-CD39-AR pathway has been proposed as a novel approach to immunotherapy, either alone or in combination with existing treatments such as PD-L1 blockade (160, 161). In mouse models, blocking CD39 has been demonstrated to reduce the tumor burden and increase infiltration of immune cells, including DCs and NK cells (161, 226). It is further reported that CD73 blockade, which has also been shown to increase the efficacy of both anti-CTLA-4 and anti-PD-L1 therapies, enhances the efficacy of radiotherapy by promoting the anti-tumor activity of the immune system (227) (228). In line with this, blocking A2AR also boosts anti-tumor activity (229). As these targets are bound to the extracellular side of the cell membrane, they can be targeted not only by small-molecule inhibitors, but also by using therapeutic antibodies, and multiple drug candidates have already entered phase I clinical trials (161). The expression of both CD39 and CD73 is enhanced by tumor-derived lactate, indicating that targeting lactate may potentiate immunotherapy through the adenosine-PKA axis (230). Indeed, Sun et al. (231) demonstrated that LDH blockade using oxamate enhanced the efficacy of CAR T cell therapy in a murine model for glioblastoma by suppressing the expression of both CD39 and CD73, highlighting the fact that targeting the TME can enhance several forms of immunotherapy.

Although PKA can also be directly targeted using small-molecule inhibitors, its widespread expression across most tissues poses a substantial challenge due to the high risk of off-target effects. This can be addressed by selectively targeting specific PKA subunits. As previously mentioned, Na et al. (170) demonstrated that the PKA subunit Cβ drives pro-tumoral function in macrophages. Notably, immune cells express a unique subunit, Cβ2, which may serve as a target in the PKA axis that will not induce systemic toxicity (171). In support of this, we recently demonstrated that tumor growth and metastasis was reduced in a murine model for metastatic breast cancer ablated for Cβ2, which was further associated with increased overall survival (169). Given that current PKA inhibitors broadly suppress all PKA activity, there is a clear need to develop novel, isoform-specific inhibitors that selectively target the Cβ variants.

## 7 The complexity of using metabolic inhibitors in therapeutic applications

Developing a drug is a lengthy and complex process typically involving several stages that include early drug identification and optimization followed by preclinical development and application for regulatory approval to initiate clinical trials (232). Once regulatory approval is granted, the compound enters clinical testing in humans, which is conducted in at least three phases. Phase I focuses on evaluating safety and determining appropriate dosage; Phase II assesses efficacy and monitors for adverse side effects; and Phase III involves large-scale trials to confirm the efficacy and safety in a broader patient population and compares the new treatment to current standard-of-care therapies (232). During this process more than 90% of preclinical drug candidates are disqualified for further development (233). Due to the complexity of metabolism, redundancies in metabolic pathways and the fact that various cells share vital metabolic features, most drugs developed to target metabolic enzymes show low efficacy or will have side effects. An example is the glutamine antagonist 6−diazo−5−oxo−L−norleucine (DON). DON, which is a non-proteogenic amino acid that blocks glutamine metabolism in all cells, resulting in severe adverse effects in patients due to distinct roles of glutamine in different cells and tissues (234, 235). Because of this, research on novel glutaminase inhibitors have led to the identification of several compounds including inhibitors BPTES which is an allosteric inhibitor with a low µM IC_50_ (236) and CB839, which is effective in the low nM range (237). Both BPTES and CB839 are glutaminase isoform type 1 specific with different mechanisms of action where CB839 appears to be well-tolerated with few side effects by patients and it is currently in several clinical trials for the treatment of various diseases including different cancers (238). (**Table 1**, https://clinicaltrials.gov/).

**Table 1:** Brief selection of inhibitors in clinical trials.

Target	Compound	Implication	Combination	Phase	Trial ID
IDO1	epacadostat	Metastatic melanoma	Pembrolizumab	III	NCT02752074
GLS1	CB839	–	–	I	NCT04607512
GLS1	CB839	Melanoma, clear cell renal cell carcinoma, non-small cell lung cancer	Nivolumab	I	NCT02771626
GLS1	CB839	Advanced stage non-small cell lung cancer	Sapanisertib	I/Ib	NCT04250545
GLS1	CB839	–	Famotidine	I	NCT04540965
PFKFB3	PFK-158	Advanced solid malignancies	–	I	NCT02044861
IDH	Ivosidenib	Glioma with advanced solid tumors	Nivolumab	II	NCT04056910
IDH	Ivosidenib	Nonresectable or Metastatic Cholangiocarcinoma	Nivolumab + Ipilimumab	I/II	NCT05921760
A2A	CPI-444	Non-small cell lung cancer	Atezolizumab	I/IIb	NCT03337698
A2A	AZD4635	Metastatic castration-resistant prostate cancer	Durvalumab	II	NCT04495179
CD73	Oleclumab	non-small-cell lung cancer	Osimertinib	I	NCT03381274
CD39	IPH5201	Advanced solid tumors	Durvalumab +/​- Oleclumab	I	NCT04261075

Extracted from https://clinicaltrials.gov/).

Furthermore, it has become clear that efficacy of drugs targeting metabolism may be limited due to the inherent flexibility, compensation and redundancies in metabolic pathways (90). As a result, even inhibitors with nanomolar affinity for their target may show limited therapeutic effect when used as monotherapies. Several examples illustrate this challenge. For instance, drugs targeting glycolytic enzymes downstream of hexokinase can be bypassed through the pentose phosphate pathway (PPP) where glucose 6-phosphate is shunted into the PPP and re-enters glycolysis as intermediates, effectively circumventing vital metabolic steps in the glycolytic pathway (239). Similarly, pyruvate from glycolysis can enter the TCA cycle via different routes depending on oxygen levels, allowing cells to maintain energy production under both aerobic and anaerobic conditions. Another example comes from the fact that pyruvate can enter the TCA cycle via different routes depending on oxygen levels in both an oxidative and energy-dependent fashion (240). In addition to this, drugs targeting LDH may simply shift metabolism towards a more oxidative phenotype and in that way be inefficient in inhibiting energy extraction in rapidly proliferating cells (90). Moreover, inhibiting glutaminase in glutamine consuming cells, may be compensated for by increased combustion of glucose, during which lack of carbons from glutamine is compensated for by glucose (88, 241). Finally, drug sensitive cancer cell lines can also develop resistance to metabolic inhibitors by rewiring their metabolic programs (90, 242–246). Together, these examples highlights the limitations of metabolic inhibitors when used alone and underscore the need for combination therapies. this stresses the complexity of metabolic inhibitors used as mono therapeutics. Due to this the use of metabolic inhibitors in conjunction with other treatments is emerging.

The rationale behind combinatorial strategies in cancer treatment includes targeting more than one metabolic process/pathway simultaneously reducing the required dose of individual agents and minimizing the risk of tumor immune evasion. Using the combination of drugs targeting metabolic pathways that support an immunosuppressive TME with ICT has been shown to further boost the anti-tumor responses of the immune system in preclinical models. In line with this, the IDH inhibitor ivosidenib is currently being tested as a candidate for combination therapy across several clinical trials in combination with PD-1 blockade (**Table 1**). There are also several non-metabolic drug candidates targeting cell surface receptors such as A2A and A2B, which have entered clinical trials in combination with PD-1 blockade, and while early reports indicate some adverse effects, including autoimmunity, these are considered manageable (247). The evidence supporting the use of inhibitors targeting glycolysis or glutamine metabolism in combination with ICT in patients is currently lacking, and hence need further exploration. At present, LDH inhibitors such as FX11, GNE-140, NCI-737, Galloflavin have also been postulated to be used to prevent tumor immune evasion when used in conjunction with PD-1 blockade (248–251). The same, but less convincing, is the case with CB839 in conjunction with PD-1 (96). In a mouse melanoma models, CB839 on its own has little effect, but when combined with anti−PD−1 but also anti−CTLA−4, it significantly suppressed tumor growth and increased infiltration of CD4^+^ and CD8^+^ T−cells (252). However, a phase I/II study of the safety and efficacy of CB839 in combination with the PD-1 inhibitor nivolumab in patients with metastatic melanoma, renal cell carcinoma, and non-small-cell lung cancer was well tolerated, but did not show increased efficacy (253). The reason for this is not known. However, the patients were not stratified based on metabolic phenotyping. As the metabolic landscape is highly variable across patients and tumors (254–256), future clinical trials should attempt to incorporate metabolic profiling to determine whether specific metabolic phenotypes correlate with improved outcomes of combination therapies.

Another obstacle in developing drugs targeting metabolism is that drugs may fail to reproduce the beneficial effect seen in preclinical models, and thus, be screened out in early clinical trials (232, 233). A striking example of this is the ECHO-301 trial, a phase III clinical trial where the IDO1 inhibitor epacadostat in combination with anti-PD1 treatment failed to provide a significantly improved patient outcome (257). However, Muller et al. (258) argues that there are several points that were inadequately discussed, which may explain the outcome, including uncertainty of whether IDO1 activity was sufficiently inhibited within the tumor, pathways bypassing IDO1 were not considered and the choice of immunotherapy over DNA damaging therapy, highlighting the need for increased understanding of metabolism within the TME. The lack of effect of a metabolic drug targeting the TME in clinical trials may be attributed to the fact that most inhibitors are screened in single cell cultures and homogenous tumor models. The latter may encompass human tumors in patient derived xenografts (PDX) animal models that may not encapsulate the complexity of tumors in the individual patient. In line with these tumors are frequently sequenced to determine patient-specific features to determine prognosis and treatment strategy. However, downstream of genetic mutations patient-specific metabolic profiles may require differential treatments despite that patients may harbor related tumors and identical oncogenic mutations. Because of this, it may be necessary to also determine metabolic phenotypes to better utilize metabolic inhibitors. Metabolic phenotypes in e.g. the TME have until now been difficult to determine. However, with extracellular flux analysis using Seahorse technology coupled with Flow cytometry has opened for more opportunities and more accurately in profiling tumor metabolic phenotypes from biopsies (259). Seahorse technology has been used to determine the metabolic phenotype of a wide array of cell types, mitochondria, 3D cell culture spheroids and now recently intact tissue biopsies (260). Seahorse profile analysis when combined with bioinformatics and artificial intelligence may in the future be useful and potential instrumental in determining combinations of drugs and treatment regimens for patient-specific targeting.

## 10 Concluding remarks

ICT is considered a game-changer in modern cancer treatment. However, favorable responses are observed in only 20-40% of patients with solid tumors (71, 72). Moreover, even when effective, current treatment strategies are often associated with a wide range of adverse effects, including liver, kidney and cardiovascular toxicity, and ICT may trigger autoimmune responses (261). These limitations highlight the need for additional therapeutic targets that can enhance anti-tumor efficacy while minimizing the side effects. Identifying targets capable of inducing synergistic or multifaceted responses might reduce the required treatment doses, thereby limit off-target toxicity while enhancing tumor clearance.

We have briefly summarized how hypoxia-driven metabolic processes in the TME contribute to the reprogramming of infiltrating immune cells and the development of a dysfunctional tumor vasculature- both of which aids cancer immune evasion and hinder effective drug delivery to the tumor. Although these factors currently pose a barrier to efficient cancer therapy, advancing our understanding of these mechanisms may enable the development of new treatment strategies for solid tumors. Given the central role of the tumor vasculature in the TME, anti-angiogenic drugs are being used in cancer therapy, and it is hypothesized that their combination with ICT may further enhance anti-tumor immune activity (262–264). However, although angiogenesis can be blocked by targeting the VEGF pathway, resistance to VEGF blockade is common (185, 187). Moreover, the combined blockade of VEGF and ICT is also correlated with adverse effects, including an increased risk of cardiovascular disease, highlighting the need for alternative therapeutic targets (265, 266). In this context, targeting metabolism in the TME might offer an alternative strategy for combination therapy. However, this approach requires the identification of metabolic targets - such as enzymes and pathways - that can be safely targeted, ideally offering synergistic effects when combined with existing therapies. Notably, the PFKFB3 inhibitor 3PO was shown to increase vessel integrity and enhance tumor perfusion, resulting in decreased hypoxia and increased drug delivery (132). As hypoxia results in upregulation of CD39 and CD73, increased tumor perfusion may also abrogate PKA-mediated immune suppression through reducing adenosine production in the TME (161). However, as PFKFB3 and glycolysis are also important in immune cell activation, it remains unclear if this would ultimately enhance or impair the efficacy of ICT (52, 104, 110, 111, 218). However, inhibition of LDH has been shown to reduce tumor growth in immunocompetent mice, but not in RAG knockout mice, indicating that inhibition of glycolysis may be beneficial in combination with ICT (249). Moreover, blocking lactate transport by targeting MCT1 and MCT4 has been associated with enhanced efficacy of PD-L1 blockade (210, 221). While this may partly stem from effects on reducing lactate-induced inhibition of T cells, reducing tumor acidification may also increase antibody affinity within the TME (210, 267). Glycolysis can further be targeted indirectly by disrupting glutamine metabolism via inhibition of the transcription factor MondoA (94). Although glutaminolysis is required for adequate T cell proliferation and cytokine secretion (126, 129), the GLS1 inhibitor CB839 - which is currently approved for phase 1B clinical trials - has shown minimal effects on CD4+ T cells at higher doses than those required for growth inhibition in cancer cells (125). Moreover, CB839 reduces endothelial cell proliferation without cytotoxic effects, as well as promoting M1-like macrophage polarization, suggesting its potential for combination with ICT to further boost anti-tumor immune responses (198). Additionally, these strategies may also be combined with drugs targeting cancer-specific mutations, including mutated IDH1 and IDH2, which are also known contributors of TME-induced immunosuppression.

This body of evidence suggests that targeting the metabolism of the TME might have synergistic effects by alleviating multiple aspects of TME-induced vascular dysfunction and immune suppression. Although this review has focused on ICT, there is evidence that these concepts are applicable to ACT as well. Future studies are needed to elucidate the synergistic potential for combining metabolic inhibition with ICT.
